# Psychometric properties of perceived barriers to PKU treatment inventory in a Brazilian national sample

**DOI:** 10.1186/s13023-025-03998-5

**Published:** 2025-09-24

**Authors:** Katia Irie Teruya, Eduardo Remor, Ida Vanessa Doederlein Schwartz

**Affiliations:** 1 Postgraduate Program in Psychology, Universidade Federal do Rio Grande do Sul, Porto Alegre, Brazil; 2 Postgraduate Program in Psychology, Institute of Psychology, Social Work, Health and Human Communication, Universidade Federal do Rio Grande do Sul, Porto Alegre, Brazil; 3 Department of Genetics, Universidade Federal do Rio Grande do Sul, Porto Alegre, Brazil; 4 Medical Genetics Service, Hospital de Clinicas de Porto Alegre, Porto Alegre, Brazil

**Keywords:** PKU, Barriers, Assessment, Treatment, Caregivers, Adherence

## Abstract

**Background:**

Metabolic control in the phenylketonuria (PKU) population is challenging. Perceived barriers to treatment adherence have been shown to be associated with metabolic control outcomes. The objective of this study was to evaluate the psychometric properties of the Perceived Barriers to PKU Treatment Inventory with patients and caregivers in different regions of Brazil.

**Methods:**

This is a cross-sectional, observational, non-probability sampling study. The inventory has 25 dichotomous items and two versions, patients and caregivers. Data collection in the present study included online assessment (*n* = 241) and presential mode (*n* = 42). Sixty-three PKU patients and 220 caregivers of individuals with PKU participated in this study. Internal consistency reliability, convergent validity, known-group validity, and discriminant validity of both versions (patient and proxy report) were evaluated. Furthermore, score norms based on the current sample are presented. A content analysis of the responses recorded in the open-ended inventory was also conducted.

**Results:**

Adequate reliability and evidence for convergent validity, known- groups validity, and discriminant validity were observed in this sample. Optimal cutoff points were identified through ROC curves (score > 9 for the patient version and score > 6 for the caregiver-proxy version), allowing for the identification of the risk of poor metabolic control due to barriers to dietary adherence. Furthermore, the qualitative analysis showed the relevance of the 25 items and the importance of addressing socio-economic and health system factors that have a significant economic impact on patients’ and families’ lives.

**Conclusion:**

The findings indicate that the Perceived Barriers to PKU Treatment Inventory is valuable for screening barriers that may contribute to increasing the difficulty of treatment management. And to identify patients and caregivers who may require targeted support to improve dietary adherence and metabolic control.

**Clinical trial number:**

Not applicable

**Supplementary Information:**

The online version contains supplementary material available at 10.1186/s13023-025-03998-5.

## Introduction

Phenylketonuria (PKU) is a rare genetic disorder that can cause motor deficits, seizures, neurodevelopmental damage, microcephaly, and intellectual disability in untreated individuals [1]. The global prevalence of PKU is estimated to be 1 in 23,930 live births [2]. In Latin America, the prevalence is approximately 1 in 23,000 [3], while in Brazil, it is around 1 in 25,000 [2]. The neurological impairments associated with the disease result from neurotoxic effects related to high blood levels of phenylalanine (Phe), an amino acid that accumulates in the body due to deficiencies in its metabolic process [4]. Treatment can prevent the manifestation of neurodevelopmental disorders in early childhood and neurocognitive function which can occur at any age in the presence of high levels of Phe [5].

Treatment for PKU includes following a Phe-free diet, using a Phe-free amino acid mixture (a medication required to replace nutrients lost due to dietary restrictions), undergoing regular blood tests to monitor plasma Phe levels, and attending medical appointments for counseling if blood tests indicate health risks [6]. Adherence to these recommendations helps maintain low plasma Phe levels, ensuring metabolic control.

Despite the health benefits of lifelong dietary treatment, many studies show low adherence among people with PKU [7–10]. In a study of adolescent and adult patients with PKU in the United Kingdom and Australia, 80% had Phe concentrations above recommended levels [11], and in the United States, an estimated 77% of adults with PKU were not keeping clinical appointments [12]. In Brazil, compliance with recommended blood Phe levels varies by patient age group: 67% up to 1 year and 63% between 1 and 4 years [13]. In the same study, the maximum limit accepted from the age of 12 years was up to 600 µmol/L (except for pregnant women), so that 48% between 18 and 29 years and 44% over 30 years were within this limit.

Treatment adherence is a complex and multifaceted behavior [14], with many influencing factors, including socioeconomic, disease-related, treatment-related, health system, and individual factors [15]. Patients with PKU and their parents/caregivers have reported a number of aspects that challenge their ability to adhere to PKU treatment adequately [16, 17]. According to the WHO [15], the importance of the role of barriers in poor patient adherence to treatment in various health contexts is well recognized, so removing barriers to adherence should be a central target of interventions aimed at improving population health.

Previously, studies had found that predictors of metabolic control included the patient’s age, as adherence deteriorated with age [8, 18, 19], lower treatment knowledge [20], lower caregiver education, and lower income [18, 21]. In addition, other factors related to metabolic control include beliefs and behaviors. For instance, greater perceived strategy effectiveness for adherence was positively associated with Phe levels, and those who used strategies rated as maladaptive had higher blood Phe levels than those who did not use them [22].

Research to identify perceived barriers to treatment is justified by the benefit to the field of studying the difficulties individuals face in achieving the best outcomes from treatment. In this sense, it can contribute to advances in studies of self-care behaviors and health prevention, which are important components of chronic disease management. In addition, programs that take into account the specific needs of each patient are more likely to be effective than a non-personalized approach [23, 24], so instruments that help identify barriers can be relevant tools to support patient-centered care. Recognizing the role of various factors in treatment adherence—especially those that may interfere with good metabolic control—the Perceived Barriers to PKU Treatment Inventory was developed [25]. To the best of our knowledge, no other scale specifically assesses barriers to PKU treatment.

The Perceived Barriers to PKU Treatment Inventory is a specific barrier instrument that is short and simple to administer to patients and their caregivers. Its results should be considered both qualitatively and quantitatively, as factors of different natures may compromise patient adherence. At the same time, the number of barriers may aggravate the attempt to improve Phe levels. Thus, the goal is to identify each patient’s difficulties in achieving better adherence so that the healthcare team can define a personalized approach tailored to their specific needs. For this reason, the Perceived Barriers to PKU Treatment Inventory helps make the process of identifying barriers more objective and standardized. It has already been used in an independent study involving patients with PKU (e.g [26]).

The previously reported evidence of validity of the instrument [25] included criterion-related validity (i.e., positive association between phenylalanine levels and the number of perceived barriers) and Known-group validity (i.e., significant difference found across age groups, with the inventory score of the adults being higher than that of the younger group). This finding was consistent with previous findings of worsening adherence in older patients, who may be experiencing an accumulation of challenges and scarce resources, leading to the perception of more barriers.

The continuous evaluation of the psychometric properties of the Perceived Barriers to PKU Treatment Inventory can provide and expand important data on the magnitude of its capacity to access the information it proposes, identifying with more precision the role of barriers in health treatment and self-care and contributing to the advancement of knowledge in the area of adherence to highly restrictive dietary regimes. This study aimed to conduct a psychometric evaluation of the Perceived Barriers to PKU Treatment Inventory in a nationwide sample.

## Methods

### Participants

A total of 63 patients with PKU and 220 caregivers were included in this study. Twenty-two patients (19 adults and three adolescents) and 20 caregivers completed the survey in person, and 41 patients (32 adults and nine adolescents) and 200 caregivers completed the survey online. During the online survey period, 27 adolescent patients, 67 adult patients, and 319 caregivers accessed the survey platform. Among those who accessed the survey platform and read the digital consent form, 16 adolescents, 23 adults, and 54 caregivers declined to participate and were subsequently directed to the acknowledgment page. After providing consent, two adolescents, 12 adult patients, and 65 caregivers were excluded from the sample because they exited the survey before completing all required questionnaires.

There was no difference in the median age between the two groups of patients (in-person and online) or in the age of the two groups of caregivers. More females with PKU responded to the online survey than males with PKU. An equal number of women and men participated in the in-person patient group. Among caregivers, women were in the majority in both types of surveys.

### Instruments

#### The perceived barriers to phenylketonuria treatment inventory

This self-administered questionnaire [25] is composed of 25 dichotomous items (“agree” or “disagree”) related to perceived barriers to the treatment of PKU. The items include aspects related to the five domains related to adherence behavior as classified by the WHO [15]. For example, item 6 refers to a socio-economic aspect (‘I don’t understand what can happen if I don’t take the treatment.’); item 9 refers to an individual aspect (‘I believe that the disease can’t harm my health.’); item 18 refers to a treatment aspect (‘I find the diet too strict. If I could eat a wider variety of foods, it would be easier.’); item 20 refers to a disease aspect (‘I don’t feel anything when I don’t follow the diet and that makes me not worry about the disease.’); and item 22 refers to a health system aspect (‘I try hard to follow the treatment, but I feel that the medical staff don’t believe me.’).

After item 25, there is also an open and optional question asking the respondent to record any other barriers to PKU treatment not mentioned in any of the previous 25 items. Respondents can select how many of the barriers apply to them. A count of the number of barriers selected results in a score, with higher scores indicating more barriers. It was available in two versions: for patients aged 13 years and over and for caregivers of pediatric patients with PKU. A paper version (for in-person participants) and a digital version (for online completion) of the instrument were used and administered to different groups of participants. Following the guidelines from the International Test Commission, researchers interested in using the inventory should contact the authors to obtain the complete version of the instrument, scoring instructions, and proper authorization for use.

#### Three single-item visual analogue scales (VAS)

Three VAS [27] were used to measure participants’ self-assessed knowledge of PKU, knowledge about PKU diet, and perceived adherence to treatment. The following questions were used to assess knowledge: “When you compare yourself to other PKU patients, how much would you say you know about the disease? (patient’s version); or “When you compare yourself to other parents of patients with PKU, how much would you say you know about the disease?” (proxies’ version). In order to assess knowledge about the diet, the question was: “When you compare yourself to other PKU patients, how much would you say you know about diet?” (patient’s version); or “When you compare yourself to the other parents of patients with PKU, how much would you say you know about diet?” (proxies’ version). The anchors for both questions were “No knowledge” and “Excellent knowledge”. The perception of adherence to PKU treatment was measured by the following question: “When you compare yourself to other PKU patients, how much would you say your behaviors are in agreement with the medical treatment prescribed?” (patient’s version); or “When you compare yourself to other parents of patients with PKU, how much would you say your behaviors (your child’s behaviors) are in agreement with the medical treatment prescribed?” (proxies’ version). The anchors were “In disagreement” and “In agreement”. The paper-based Visual Analog Scales (VAS) featured three 10 cm horizontal lines, where respondents marked their answers with a pen. In the digital version, these lines were replaced by a movable cursor. The anchors remained the same for both formats. Respondents who completed the online Perceived Barriers to Phenylketonuria Treatment Inventory also used the online VAS, while those who used the paper-based inventory completed the paper VAS.

### Procedures

This study employed a cross-sectional, observational design with non-probability sampling to evaluate the psychometric properties of a self-report questionnaire assessing perceived barriers to treatment in individuals diagnosed with phenylketonuria (PKU) and adults responsible for managing PKU in a family member. Data collection was conducted through both in-person and online surveys. All participants were volunteers who provided informed consent prior to participation. The data were anonymized and processed in accordance with the Brazilian General Data Protection Law. No monetary compensation was offered to participants.

#### In person data collection

Patients aged 12 years and older with Phenylketonuria (PKU), along with caregivers of pediatric patients, were recruited from the medical genetics service of a university hospital in southern Brazil. Individuals with severe or disabling chronic diseases unrelated to PKU, as well as caregivers of such patients, were excluded from the study. All eligible patients and caregivers who attended medical and nutritional appointments during the study period were invited to participate. Data from in-person participants were collected between October 2022 and February 2024.

#### Online data collection

A digital assessment protocol version was made available through the web-based data management platform Survey Monkey™, which included a sociodemographic questionnaire and the Perceived Barriers to PKU Treatment Inventory [25]. The digital assessment protocol, which was designed to be anonymous to protect participant privacy, has three versions - adult patient (over 18 years old), adolescent patient (12 to 17 years old), and caregiver. Respondents were asked to choose one of three options, which guided them to the appropriate informed consent form. In the case of the adolescent patient version, parents or guardians were required to authorize and agree to the adolescent’s participation. The informed consent form outlined the purpose and significance of the study, emphasizing that participants could withdraw at any time. It also assured participants of their anonymity.

Participants were recruited through social media (Instagram profiles) followed by patients with PKU, parents/caregivers and healthcare professionals. The research was also promoted on a social media profile developed during the study, focusing on evidence-based self-care related to PKU (https://www.instagram.com/autocuidado.e.pku/). Reference centres for neonatal/genetic screening across the country were contacted by one of the researchers via a telephone call to ask for their support in disseminating the survey to the PKU patients who were being treated at these facilities. Exclusion criteria: individuals without access to an electronic device (e.g. PC, tablet or cell phone) with an Internet connection to access and answer the questionnaires. Online data was collected between August 2023 and February 2024.

### Data analysis

#### Quantitative analyses

Descriptive statistics for the continuous (median and interquartile range) and categorical data (absolute and relative frequency) were calculated. The Mann-Whitney test was used to compare groups in continuous variables, Fisher’s exact test was used to compare groups in categorical variables, and Kendall’s Tau b was used for correlation analysis. The internal consistency for dichotomous items was measured by Kuder-Richardson Formula 20 (KR-20; [27]). Evidences of validity was assessed through convergent validity and known groups validity. Convergent validity was evaluated by correlating the scores from the Perceived Barriers to PKU Treatment Inventory with the three measures obtained from the Visual Analogue Scales. The validity of known groups was assessed by comparing two age groups (one represented by adult patients and the other by caregivers as proxies for patients under 18). Furthermore, score norms (i.e., percentiles) based on the current sample are presented. All quantitative analyses described above were performed using SPSS 18 version software (SPSS, Inc.).

With the data collected in-person in the medical genetics service, Receiver Operating Characteristic (ROC) curve analyses were conducted using Jamovi Computer Software (version 2.6) to establish the optimal cutoff points and discriminatory properties of the inventory protocol in detecting poor metabolic control (determined by median Phe levels in the past year. Poor metabolic control equals Phe levels > 6 mg/dl in patients up to 12 years old and > 10 mg/dl in patients aged 12 years or older [26]). The optimal cutoff points were chosen by Youden Index. We plotted a ROC curve (graph of sensitivity/1 − specificity for the inventory scores against adherence level) and examined the area under the curve (AUC); an AUC of 1.0 corresponds to a perfect test with 100% sensitivity and 100% specificity, whereas an area of 0.5 represents random discrimination. We calculated positive and negative predictive values (PPVs and NPVs) for the chosen cutoff scores.

#### Qualitative analyses

The relevance of the content of each of the 25 dichotomous items was examined by determining how often both groups agreed with the item. The responses to the qualitative item of the instrument were analyzed using content analysis (deductive approach) and presented in the supplementary material.

## Results

In all four groups, at least 90% of respondents said that the diagnosis was made at newborn screening. Patients in-person and online showed differences related to gender (χ^2^ = 5.198, *df* = 1, *p* = .023) and ethnicity (χ2 = 4.611, *df* = 1, *p* = .032). In addition, when compared to online patients, in-person patients were older (*U* = 402,000, *p* = .50, *r*_*rb*_ = 0.107), and reported a lower number of barriers (*U* = 276,000, *p* = .012, *r*_*rb*_ = -0.388). When compared in-person and online caregivers, there were differences in ethnicity (χ^2^ = 5.862, *df* = 1, *p* = .015), type of PKU (χ^2^ = 7.803, *df* = 3, *p* = .050), and number of barriers reported (*U* = 756,000, *p* = < 0.001, *r*_*rb*_ = -0.622), and online group reported significantly more barriers. The demographic characteristics and instruments results of participants are shown in Table 1.

**Table 1:** Demographic characteristics and instruments results of the participants

	Caregivers In-person (*n* = 20)	Caregivers Online (*n* = 200)	Statisticsp-value	Patients In-person (*n* = 22)	Patients Online (*n* = 41)	Statistics *p*-value	
**Age** Median (IQR)	40.0 (36.5–42.5)	37.0 (33.0–42.0)	0.223	24.5 (22–30)	24.0 (20–29)	0.050	
**Ages of the children with PKU that the caregiver supports.** Median (IQR)	7.5 (5–11)	10 (5–15)	0.161	na	na		
**Sex**			0.243			0.023	
Female	17 (85%)	185 (92.5%)		11 (50%)	32 (78%)		
Male	3 (15%)	15 (7.5%)		11 (50%)	9 (22%)		
**Ethnicity**			0.015			0.032	
White	18 (90%)	126 (63%)		21 (95,4%)	30 (73,2%)		
Non-white	2 (10%)	74 (37%)		1 (4,5%)	11 (26,8%)		
**Education** ^**a**^			0.172			0.086	
Up to 4 years	6 (30%)	31 (15.5%)		5 (23%)	6 (14.7%)		
5–11 years	7 (35%)	76 (38%)		13 (59%)	16 (39%)		
≥ 12 years	6 (30%)	93 (46.5%)		4 (18%)	19 (46.3%)		
Not reported	1 (5%)	0		0	0		
**The caregiver is responsible for**			0.519				
A female patient	10 (50%)	115 (57.5%)		na	na		
A male patient	10 (50%)	85 (42.5%)					
**PKU type**			0.050			0.191	
Classic	9 (45%)	61 (30.5%)		12(54.5%)	15 (36.5%)		
Mild	11 (55%)	23 (11,5%)		10 (45.5%)	6 (14.6%)		
I don’t know	0	8 (4%)		0	2 (4.9%)		
Not reported	0	108 (54%)		0	18 (44%)		
**PKU diagnosis was established**			0.942			0.649	
Through Newborn Screening (NBS)	18 (90%)	181 (90.5%)		21(95.5%)	40 (97.5%)		
Later on NBS	2 (10%)	18 (9%)		1 (4.5%)	1 (2.5%)		
I don’t know	0	1 (0.5%)		0	0		
**Perceived Barriers to Phenylketonuria Treatment Inventory** Median (IQR)	3.5 (2–4.2)	7 (4.7–12)	< 0.001	5 (3.2–9.7)	10 (6–12)	0.012	
**VAS –** **disease knowledge** Median (IQR)	69 (60.2–82)	64 (49–80)	0.351	65 (58–74.5)	72 (55–89)	0.161	
**VAS –diet knowledge** Median (IQR)	71.5 (58–89.7)	70.5 (49–85)	0.411	82 (63.5–93.5)	82 (62–100)	0.694	
**VAS – adherence** Median (IQR)	81 (64.7–89.2)	79.5 (59.7–98.2)	0.749	70 (49.5–76)	76 (53–90)	0.212	

*Note*. a = In the patient group, only the educational level of the adults was included because the adolescents were still in school. *Abbreviations*: IQR = Interquartile range; PKU = Phenylketonuria; na = not appliable; VAS = Visual Analogue Scale. The Mann-Whitney test was used to compare groups in continuous variables; Fisher’s exact test was used to compare groups in categorical variables

A comparison of caregivers in the southernmost state of the country was conducted between those who responded in person (*n* = 20) and those who responded online (*n* = 28). The in-person group reported fewer barriers (Median = 3.50; IQR = 2.25) than the online group (Median = 4.5; IQR = 6.5), a difference that was found to be statistically significant (U = 176.00; *p* = .028; *r*_*rb*_ = -0.371). To eliminate the potential bias introduced by the response method and ensure that any observed differences were more likely due to geographical location rather than a confounding variable, only the online respondents were included in a subsequent descriptive analysis by region. This decision, justified by the initial finding of a significant difference between the two response methods, allows for a more accurate exploration of geographical variations in caregiver barriers. The number of participants and the median number of barriers for each area are provided in Table 2.

**Table 2:** Descriptive statistics of perceived barriers to treatment from online respondents, for caregivers and patients with PKU, categorized by region of Brazil

Regions of Brazil	Patients with PKU *n* (%)	Patients’ score Mdn (IQR)	Caregivers *n* (%)	Caregivers’ score Mdn (IQR)
Southeastern	12 (29.2)	6.5 (6)	64 (32.0)	8 (7)
Southern	11 (26.8)	10 (6)	52 (26.0)	6 (6)
Northeastern	9 (21.9)	10 (12)	35 (17.5)	11 (6)
Midwestern	7 (17.0)	10 (9)	34 (17.0)	5.5 (8)
Northern	2 (4.8)	12 (*)	15 (7.5)	10 (8)
Total	41 (100)	10 (6)	200 (100)	7 (8)

*Note: Mdn = Median*,* IQR = Interquartile Range*, *With only two values, quartiles can’t be defined properly

Among patients, there were no significant differences in the number of reported barriers across the five regions (χ²(4) = 4.287; *p* = .369). In contrast, caregivers reported significant regional differences (χ²(4) = 20.567; *p* < .001). The Southern region had fewer barriers than both the Northeastern (U = 470,000; *p* < .001) and Northern regions (U = 199,000; *p* = .004), while the Midwestern region reported fewer barriers than the Northeastern region (U = 351,000; *p* = .003).

### Reliability

The internal consistency coefficients for the Perceived Barriers to Phenylketonuria Treatment Inventory were as follows: in-person patients (*n* = 22, KR20 = 0.80), online patients (*n* = 41, KR20 = 0.77), in-person caregivers (*n* = 20, KR20 = 0.67), and online caregivers (*n* = 200, KR20 = 0.82).

### Evidences of validity: convergent validity

Previous studies suggest that patients’ [28] and caregivers’ [20, 29] knowledge of the disease and treatment affects adherence. Greater awareness can help address individual, social, and treatment-related challenges, leading to fewer perceived barriers. If the instrument effectively captures these barriers, its results should reflect how far adherence deviates from the ideal, as the Visual Analogue Scale (VAS) indicates.

This study found a negative correlation between the VAS measures for online patients and caregivers and the barriers inventory scores. In-person patients showed a negative association between the number of barriers and the VAS scores for disease knowledge and adherence. For in-person caregivers, only the VAS score related to diet knowledge was linked to the number of barriers (see Table 3).

**Table 3:** Correlations between the scores of the perceived barriers to PKU treatment inventory and the three visual analogue scales (VAS) of patients and caregivers in the in-person and online survey

	Patients In-person (*n* = 19)*	Caregivers In-person (*n* = 20)	Patients Online (*n* = 41)	Caregivers Online (*n* = 200)
	Perceived Barriers to PKU Treatment Inventory (Scores)			
VAS – disease knowledge	− 0.467 *p* = .007	− 0.247 *p* = .146	− 0.261 *p* = .022	− 0.299 *p* = .000
VAS – diet knowledge	− 0.012 *p* = .994	− 0.407 *p* = .016	− 0.385 *p* = .001	− 0.304 *p* = .000
VAS – adherence	− 0.493 *p* = .004	− 0.017 *p* = .921	− 0.335 *p* = .003	− 0.310 *p* = .000

*Note*. * Three patients did not responded to Visual Analogue Scale (VAS). Kendall’Tau b test was used to conduct correlations

### Evidences of validity: known group validity

The analysis aimed to evaluate the instrument’s effectiveness in identifying barriers throughout the lifespan, noting that adults with phenylketonuria (PKU) tend to have higher phenylalanine (Phe) levels than younger patients. It is expected that as patients reach adulthood, the percentage with good adherence will decline, indicating more barriers after childhood. In the present study, related to the in-person groups, when adult patients (*Mdn* = 5.0, *IQR* = 6.5) were compared to younger (*Mdn* = 3.5, *IQR* = 2.25; responded by their proxy caregivers) a significant difference was found in the score of the Perceived Barriers to Phenylketonuria Treatment Inventory (*U* = 113.500 *p* = .031) showing the perception of more challenges among adults. On the other hand, when considering online respondents, there was no difference in the total score of answers of adults with PKU (*Mdn* = 10.0, *IQR* = 6.0) and youngers (*Mdn* = 7.0, *IQR* = 7.25; answered by caregivers) (*U* = 2539,000, *p* = .060).

### Evidences of validity: discriminant validity

ROC curve analyses were performed to evaluate the discriminant validity of the Perceived Barriers to PKU Treatment Inventory to detect risk for poor metabolic control. We used the data collected in person in the medical genetics service. This set of data includes phenylalanine levels recorded in medical reports. The median Phe level over the 12 months prior to participation in the study was used to classify participants as having good or poor metabolic control.

**Fig. 1:**
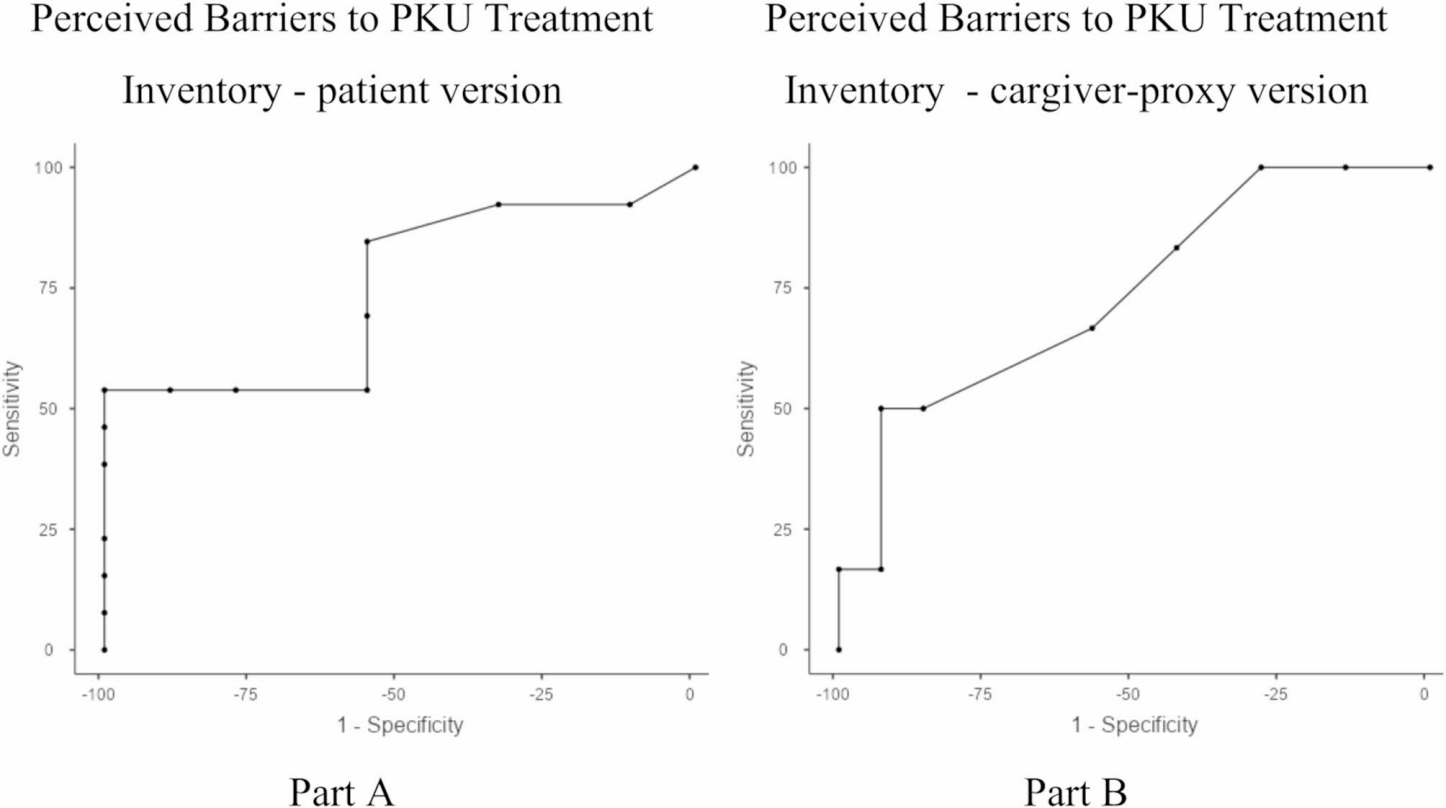
Perceived Barriers to PKU Treatment Inventory ROC curves for detecting non-adherence related to PKU treatment

Combining the information in Table 4; Fig. 1 shows that the Perceived Barriers to PKU Treatment Inventory, patient (Part A; AUC = 0.75) and caregiver-proxy (Part B; AUC = 0.74) versions, had statistically adequate AUC for detecting patients with poor metabolic control. Table 4 shows the discriminatory properties of the inventory for predicting poor metabolic control. The optimal cutoff points for patients (cutoff 9) and caregiver-proxy version (cutoff 6) were chosen based on Youden’s index. According to the results, patients reporting scores above nine may have a risk of poor metabolic control due to low adherence to diet. On the other hand, caregivers answering a proxy version with scores above six may indicate their child is not achieving the desired level of phenylalanine control through diet.

**Table 4:** Discriminant indices of perceived barriers to PKU treatment inventory in detecting non-adherence related to PKU treatment

Perceived Barriers to PKU Treatment Inventory	Optimal Cutoff point	Sensitivity (%)	Specificity (%)	PPV (%)	NPV (%)	Youden’s index	AUC
Patient version (*n* = 22)	9	53.85%	100%	100%	60%	0.538	0.748
Cargiver version (*n* = 20)	6	50%	92.86%	75%	81.25%	0.429	0.738

PPV positive predicted value, NPV negative predicted value, AUC area under the curve

### Norms for the perceived barriers to phenylketonuria treatment inventory

Based on the patient and caregiver scores from the Brazilian in-person and online sample, the 25th, 50th, 75th, and 85th percentiles of the score’s inventory were calculated (see Table 5).

**Table 5:** Percentile values for the perceived barriers to PKU treatment inventory calculated from the responses of the sample of patients and caregivers. Brazil, 2024

	Scores (Frequency of perceived barriers)					
	Total		In-Person		Online	
	Patients (*n* = 63)	Caregivers (*n* = 220)	Patients (*n* = 22)	Caregivers (*n* = 20)	Patients (*n* = 41)	Caregivers (*n* = 200)
Mean	8.55	7.84	6.54	3.65	9.63	8.27
*SD*	4.57	4.64	4.25	2.75	4.42	4.59
Median	9.00	7.00	5.00	3.50	10.00	7.00
*IQR*	6.50	7.00	6.50	2.75	6.00	7.75
Percentiles						
25th	5.00	4.00	3.25	2.00	6.00	4.25
50th	9.00	7.00	5.00	3.50	10.00	7.00
75th	11.50	11.00	9.75	4.25	12.00	12.00
85th	13.70	13.00	11.00	6.00	14.00	13.00

### Frequency of dichotomous items

In the group of 63 patients with PKU who completed the instrument, the items with the lowest and highest frequency were, respectively, item 9 (“I believe that the disease cannot harm my health.” = 9.5%) and item 17 (“It would be easier to take the formula if it tasted better.” = 84.1%). When considering caregivers, the lowest level of endorsement was for item 3 (“Sometimes I’m embarrassed to tell people that he/she has the disease.” = 6.4%); on the other hand, 88.2% of the 220 respondents endorsed item 18 (“I find the diet very strict. If he/she could eat more different kinds of food, it would be easier.”). Items 17 and 18 were pertinent in both groups, as item 18 was the second most chosen item by patients, reaching 76.2% of respondents, and item 17 was indicated by 86.4% of caregivers. The percentage of patients and caregivers who participated in this study and endorsed each item, either in person or online, is shown in Table 6.

**Table 6:** Frequency of respondents (patients and caregivers) who endorsed each of the 25 items on the perceived barriers to PKU treatment inventory

Patient version of the instrument	Caregiver version of the instrument	Patients agreement n (%). *N* = 63	Caregivers agreement n (%). *N* = 220
1. I am afraid that people will treat me differently or reject me if they know that I have the disease.	1. I am afraid that people will treat my child differently or reject him/her if they know that he/she has the disease.	20 (31.7%)	120 (54.5%)
2. Sometimes I hide from people that I have the disease.	2. Sometimes I hide from people that my child has PKU.	26 (41.3%)	52 (23.6%)
3. Sometimes I feel ashamed to tell people I have the disease.	3. Sometimes I feel ashamed to tell people my kid has PKU.	23 (36.5%)	14 (6.4%)
4. Although I feel like going, I don’t go to some parties or family events because I know that there will be food that I cannot eat.	4. Although we want to go to a party or family event, we don’t go because we know that there will be food that he/she cannot eat.	21 (33.3%)	65 (33.3%)
5. I have questions about what the disease is, what causes it, how it can harm me.	5. I have questions about what the disease is, what causes it, how it can harm him/her.	10 (15.9%)	50 (22.7%)
6. I do not understand what can happen if I do not get the treatment.	6. I do not understand what can happen if my child kid doesn’t follow the treatment.	10 (15.9%)	33 (15%)
7. I have questions about what I can eat, which food is forbidden, which one is allowed and how to control the diet.	7. I have questions about what my child can eat, which food is forbidden, which one is allowed and how to control the diet.	11 (17.5%)	51 (23.2%)
8. I often have no desire to follow the diet.	8. I frequently realize that he/she doesn’t want to follow the diet.	37 (58.7%)	92 (41.8%)
9. I believe that the disease can not harm my health.	9. I believe that the disease can not harm his/her health.	6 (9.5%)	36 (16.4%)
10. I feel that I do not have people to count on to help me follow the treatment.	10. I feel that I don’t have people to count on to help me with my child’s treatment.	13 (20.6%)	76 (34.5%)
11. People in my family say different things about the treatment and I do not know what to do.	11. Sometimes I realize we say different things about the treatment to him/her and we don’t know what to do.	9 (14.3%)	70 (31.8%)
12. I frequently have no time to prepare my meals.	12. I frequently have no time to prepare his/her meals.	27 (42.9%)	81 (36.8%)
13. Planning and preparing meals take a lot of my time on a daily basis and so I cannot fully follow the diet.	13. Planning and preparing meals takes a lot of my time on a daily basis and so I cannot make my child fully follow the diet.	27 (42.9%)	54 (24.5%)
14. I get a bit confused when it comes to deciding what to cook, what ingredients I can use.	14. I get a little bit confused when it comes to deciding what to cook, what ingredients I can use.	18 (28.6%)	80 (36.4%)
15. I get a little confused when I go to a restaurant. I do not know what I can order.	15. I get a little confused when we go to a restaurant. I do not know what we can order.	19 (30.2%)	76 (34.5%)
16. Sometimes I cannot resist and I eat foods I know are forbidden.	16. Sometimes I cannot resist and allow him/her to eat foods I know are not allowed.	30 (47.6%)	49 (22.3%)
17. It would be easier to take the formula if it had a better taste.	17. It would be easier to make him/her ingest the formula if it had a better taste.	53 (84.1%)	190 (86.4%)
18. I think the diet is very strict. If I could eat a broader range of food, it would be easier.	18. I think the diet is very strict. If he/she could eat a broader range of food, it would be easier.	48 (76.2%)	194 (88.2%)
19. I do not think I have a disease. I just need to take care of my meals.	19. I do not think my kid has a disease. He/ she just needs to take care of what he/she eats.	25 (39.7%)	69 (31.4%)
20. I do not feel anything when I don’t follow the diet and that makes me not worry about the disease.	20. I can’t see any difference in my child when he/she doesn’t follow the diet and that makes me not to worry about the disease.	20 (31.7%)	13 (5.9%)
21. I notice that the medical staff treats me differently, as if I might not be able to follow the treatment.	21. I notice that the medical staff treats us differently, as if my child and I might not be able to follow the treatment.	7 (11.1%)	23 (10.5%)
22. I struggle to follow the treatment, but I feel that the medical team does not believe it.	22. We struggle to follow the treatment, but I feel that the medical team does not believe us.	10 (15.9%)	38 (17.3%)
23. I feel physicians do not believe what I say and think I do not follow what they instruct me to do.	23. I feel physicians do not believe what we say and they think we do not follow what they instruct us to do.	15 (23.8%)	48 (21.8%)
24. The distance from my house to the hospital makes it more difficult to attend the medical appointments.	24. The distance from our house to the hospital makes it more difficult to attend the medical appointments.	32 (50.8%)	82 (37.3%)
25. There are months when the formula is not delivered.	25. There are months when the formula is not delivered.	22 (34.9%)	70 (31.8%)

Note. The items were translated from the Brazilian Portuguese to English to the present paper

### Results of the open-ended question item of the perceived barriers to PKU treatment inventory

Responses to the open-ended question item of the Perceived Barriers to PKU Treatment Inventory (i.e., Make a list of up to five barriers - starting with the most important - that are most hindering your adherence to treatment that have not been mentioned previously), bring up to 801 original responses recorded.

Pre-defined categories were applied to analyze the content of the qualitative responses (deductive approach) based on the WHO’s five dimensions [15] as a theoretical reference. The qualitative analysis of responses allowed us to organize them into thematic categories by the purpose of the instrument, i.e., to identify barriers to adherence behavior. All themes, sub-themes, the definition of each cluster, and the percentage of responses categorized in each dimension are described in Tables S1 and S2 (supplementary material). Among respondents who completed the instrument online, 65.8% of patients and 83.5% of caregivers provided between one and five responses. Among those who completed the instrument in person, a lower percentage of patients and caregivers recorded answers to the open-ended question, 36.3% and 55%, respectively. Combining online and in-person responses, 108 qualitative statements were provided by patients and 693 by caregivers.

In the categorization, we found a similarity in the content of the barriers reported by patient and caregiver groups. The analysis of adolescent and adult patients with PKU responses on barriers suggested that 42.6% of the patients’ responses were related to socioeconomic factors, 20.4% to team and health system factors, 23.1% to treatment factors, and 9.25% to individual factors (See Table S1, supplementary material). There were no responses that were considered to be related to the dimension of disease factors. And the analysis of the parent’s/caregivers’ responses suggested that 40.4% were related to socioeconomic factors, 25.1% to team and health system factors, 24.5% to treatment factors, and 7.5% to individual factors (See Table S2, supplementary material).

The reader can find a complete description of the qualitative analysis reported in the supplementary material file.

### Intersection between the 25 dichotomous items and the answers to the open question in the perceived barriers to PKU treatment inventory

The optional instrument item captured barriers not covered by its 25 dichotomous items. Some responses reiterated covered topics, like treatment center distance (item 24) and social support (item 10). Others highlighted gaps, such as limited access to low-protein foods, inadequate PKU dietary options, and public awareness, are concerns that may require systemic changes (outside of the individual’s control). The complete qualitative analysis with themes raised in the responses and the corresponding items in the inventory that deal with similar issues are shown in supplementary material in Table S3.

## Discussion

This study reported new results on the psychometric properties of Perceived Barriers to PKU Treatment Inventory - patients and caregivers/proxy versions. To our knowledge, this is the first study to include representatives of PKU patients and caregivers of PKU patients from 23 out of 26 Brazilian states.

Unexpectedly, we found that the mode of response - in-person or online, affected the number of barriers reported by participants living in the same state, and one hypothesis for this finding may be the social desirability bias. Social desirability bias [30] refers to participants’ tendency to over-report socially acceptable characteristics and to deny or under-report socially undesirable characteristics or behaviors, which may have contributed to patients evaluated face-to-face (in-person) reporting fewer difficulties in managing treatment. In the present study, for example, while 15% of in-person caregivers agreed with item 8 (“*I frequently realize that he/she doesn’t want to follow the diet*.”) in the group of caregivers who responded online, almost 50% of the sample endorsed the item. Among patients, 4.5% of the in-person group reported item 6 (“*I do not understand what can happen if I do not get the treatment*.”), this percentage increased to 22% in the online group. Seeing those results, we hypothesize that the impersonality of responding online may encourage patients to be more open about their difficulties and perceived barriers. Another hypothesis that could explain the differences in scores between in-person and online assessments would be the participants’ sociodemographic characteristics. For example, when considering participants from all regions of the country, there were more female than male patients and more ethnic diversity in the online group compared to the in-person group. Furthermore, the socioeconomic and cultural context resulting from participants from different states in the online survey may have contributed to these results, as the in-person group consisted only of people from the same state.

The instrument’s reliability applied to the patients and caregivers in this study was considered good, as it ranged between 0.70 and < 0.90 [31]. As expected, associations were found between the three VAS measures and the inventory score in online groups, supporting convergent validity. The association found indicates the congruence of the items of the instrument with challenges related to adherence. Not all associations were significant for the data collected in-person, and this result may be related to the smaller sample size of the in-person group, which may have influenced the variability of responses. In terms of known-group validity, the data collected in person indicates a difference in the number of barriers faced by two age groups. This suggests that the instrument is effective in capturing the greater difficulties that adult patients experience in managing their treatment, as outlined in the literature.

In addition, the study provides evidence of discriminant validity for the Perceived Barriers to PKU Treatment Inventory in detecting risk for poor metabolic control among patients with PKU. The use of ROC curve analyses, which are widely recognized for evaluating the diagnostic accuracy of assessment tools, is a robust method for this purpose. The results demonstrate that both the patient version (AUC = 0.75) and the caregiver-proxy version (AUC = 0.74) of the inventory exhibit statistically adequate discriminatory power. According to established benchmarks, AUC values between 0.70 and 0.80 indicate acceptable accuracy, supporting the utility of the inventory as a screening tool. The cutoff points identified through Youden’s index (patient version: score > 9; caregiver-proxy version: score > 6) further enhance the practical application of the inventory. These thresholds provide actionable guidance for clinicians, suggesting that patients scoring above the cutoff may be at risk of poor metabolic control due to barriers to dietary adherence. Similarly, caregiver-proxy scores above the threshold may signal insufficient phenylalanine control in the child, highlighting areas requiring intervention. It is particularly noteworthy that the study integrates data from clinical records (e.g., median phenylalanine levels over 12 months) to establish a criterion for metabolic control. This methodological rigor strengthens the validity argument, as it ensures that the inventory’s scores are meaningfully linked to a clinically significant outcome.

Three key points emerge from the responses to both the dichotomous items and the open-ended question: (a) the relevance of the themes to the target population; (b) the perceived burden of specific barriers; and (c) the role of open-ended questions in identifying additional treatment barriers related to patients’ social contexts. When considering the burdens associated with various types of barriers, participants most frequently cited the low palatability of the amino acid mixture and the high level of food restriction as significant challenges. These findings support several previous studies that have explored factors related to patients’ metabolic control (e.g [17, 22, 28, 32–40]). Additionally, distance to the service center, which was examined through a dichotomous question and mentioned in open-ended responses, was identified as a factor that could negatively impact scheduling. This is consistent with findings from other studies [41–43].

Analyzing the qualitative responses not covered by dichotomous questions reveals a common theme: the relationship between social systems and health resources. Respondents frequently cited barriers such as high costs and limited availability of low-Phe foods, as well as inadequate social support and food options in restaurants. The economic burden of the PKU diet, especially concerning specialty low-protein products, significantly impacts metabolic control [44] and has been noted in various countries [42, 45–48]. Financial support for special foods varies by country, ranging from full state coverage to no support at all [45]. In Brazil, accessing these foods requires a court order, which is often a complex and inaccessible process for many families in need.

The population identified and reported challenges related to the need for a multidisciplinary healthcare team in the open-ended question. European guidelines [49] on best practices for managing phenylketonuria (PKU) stress the inclusion of pediatricians, nutritionists, psychologists, and clinical geneticists. Ongoing treatment requires adaptation to the patient’s physical, cognitive, and psychosocial development since diagnosis. Each developmental stage brings unique challenges [50, 51], making the support of a multidisciplinary team essential. Treatment must evolve to meet the individual’s changing needs, from starting school and forming social connections to adolescence, achieving independence, and transitioning into adulthood.

Fear, sadness, guilt, and feelings of being overwhelmed were all highlighted in the carers’ group. In addition to managing their own emotions, caregivers also have the responsibility of serving as parents and addressing their children’s feelings regarding the disease and treatment. While these topics also apply to parents, maintaining the same 25 items in both the patient and carer versions of the inventory offers a distinct advantage. This consistency allows for comparisons between the scores of patients and caregivers, enabling the monitoring of the same barriers at various stages of the patient’s development. Consequently, it becomes possible to identify which barriers persist over the years and to observe the changes that occur during the transition of responsibility for treatment when the patient reaches adulthood.

### Limitations of the study

Most of the data collected for this study came from an online survey. While this approach allowed us to gather responses from 23 out of 26 Brazilian states, it may have resulted in a biased profile of respondents. This limitation arises because the survey only reached individuals with access to internet-connected devices, thereby excluding lower-income populations and those living in areas with poor connectivity.

## Conclusion

The results of the present study suggest that the Perceived Barriers to PKU Treatment Inventory can identify issues relevant to the target population (i.e., barriers that may contribute to increasing the difficulty of treatment management). Furthermore, the instrument is valuable for identifying patients and caregivers who may require targeted support to improve dietary adherence and metabolic control. These results have important implications for clinical practice, as they provide a basis for proactive interventions to optimize health outcomes in individuals with PKU.

## Supplementary Information

Below is the link to the electronic supplementary material.

Supplementary Material 1

## Data Availability

The data that support the findings of this study are available from the corresponding author upon reasonable request.

## Abbreviations

PKU: Phenylketonuria
Phe: Phenylalanine
VAS: Visual analogue scale
ROC: Receiver operating characteristic curve
AUC: Area under the curve
PPVs: Positive predictive values
NPVs: Negative predictive values
WHO: World health organization
