# Supplementary material for: Psychometric properties of perceived barriers to PKU treatment inventory in a Brazilian national sample
Source: Orphanet J Rare Dis. 2025 Sep 24;20:487. doi: 10.1186/s13023-025-03998-5 (PMC12461978; doi:10.1186/s13023-025-03998-5)
Supplement: Supplementary file 1 — Supplementary Material 1 [file 13023_2025_3998_MOESM1_ESM.docx]

**Supplementary Material**

**Psychometric properties of Perceived Barriers to PKU Treatment Inventory in a Brazilian national sample.**

**Results of the open-ended question item of the Perceived Barriers to PKU Treatment Inventory**

In order to analyze the content of the qualitative results, pre-defined categories were applied, based on the WHO's five dimensions [15] as a theoretical reference. This allowed the material emerging from the sentences recorded in the open-ended question to be understood in accordance with the purpose of the instrument, i.e., to identify barriers to adherence behavior. The result was a reduction of the data and their organization into thematic categories based on two key processes: coding and categorizing the content of participants' responses. Themes and, where appropriate, sub-themes were then formulated to form different groups and reviewed by a second researcher.

Among respondents who completed the instrument online, 65.8% of patients and 83.5% of caregivers provided between one and five responses (five was the maximum number of responses allowed). Among those who completed the instrument in person, a lower percentage of patients and caregivers recorded answers to the open-ended question, 36.3% and 55%, respectively. Combining online and in-person responses, 108 qualitative statements were provided by patients and 693 by caregivers.

A similarity in the content reported by patient and caregiver groups was found in the categorization of barriers mentioned. For example, socioeconomic factors contributed significantly to the total number of categorized responses. Lack of access to low-Phe foods, either due to high cost or difficulty in finding them in grocery stores, and lack of social support and lack of suitable meals for PKU in restaurants were the most recurring perceived barriers for these respondents. Other topics common to both groups: distance between the patient's home to the treatment center; a perceived lack of knowledge about the disease among health professionals; difficulties in receiving the amino acid mixture on a regular basis and its poor palatability; a lack of awareness about the disease among the general population; a lack of government support for access to manufactured food products with low Phe content.

Also mentioned by both groups: other forms of treatment, such as drugs that increase the patient's tolerance to Phe; perceived high level of dietary restriction; lack of information about the disease and difficulties in preparing meals that fit the diet. Similarly, patients and caregivers did not mention responses that could be attributed to the dimension of factors related to the disease, with the exception of a single mention of damage to health related to delays in the child's development.

At the same time, some issues were mentioned only by the caregiver group and were consistent with the parents’ perspective. For example, self-blame, overwhelm, difficulties in dealing with the child's resistance to the diet, and the perception of challenges that accompany the developmental stages of their son or daughter with PKU. All themes, sub-themes, the definition of each cluster, and the percentage of responses categorized in each dimension are described in Tables S1 and S2.

Table S1. Qualitative analysis of the 108 statements of adolescents and adult patients with PKU.

| **Dimensions according to WHO (2003)** | **Themes** | **Definition** | **Frequency**  **n (%)** |
| --- | --- | --- | --- |
| **1.**  **Socio-economic related factors** | **Distance to treatment center** | Mention of distance, need for transportation.  Example: "The distance between my home and the hospital." | 6 (5.5%) |
|  | **Financial barriers** | Mention of costs associated with treatment. Example: “High cost of low-protein diet.” | 11 (10.1%) |
|  | **Difficulty in finding foods for the diet or a lack of variety in foods** | Mention of difficulties in finding diet foods in markets or perception of few options available for purchase. Example: "Having few options for processed foods.” | 18 (16.6%) |
|  | **Lack of social support from the community** | Mention of lack of support from close people;  lack of places that offer meals with options that accommodate dietary restrictions. Example: “School snacks without adaptation.” | 11 (10.1%) |
| **2.**  **Factors related to the Team and the Health System** | **Healthcare team support** | Mention of lack of knowledge about the disease; lack of more professionals to provide care; communication problems between the patient and healthcare professionals. Example: "Lack of knowledge on the part of the health care team." | 5 (4.6%) |
|  | **Problems getting amino acid mixture free of charge** | Difficulty getting the medical food regularly. Example: “Regularization of the supply of amino acid mixture.” | 6 (5.5%) |
|  | **Information provided to the public** | Perception of lack of disease information for general public. Example: "Same frequent doubts about the disease, due to lack of knowledge that is not passed on in schools/places, so the disease is barely known." | 5 (4.6%) |
|  | **Government support** | Mention of the need to improve public support in financial and patient care processes. Example: “Lack of low-protein foods available free of charge” | 6 (5.5%) |
| **3. Disease- related factors** | - | - | - |
| **4. Treatment-related factors** | **Lack of access to other resources that would help with treatment** | Mention lack of new treatment options; lack of other tools to measure Phe levels. Example: "Lack of medications that increase food tolerance." | 5 (4.6 %) |
|  | **Difficulty in consuming the amino acid mixture** | Mention of low palatability and low quality of the provided medical food. Example: "The terrible taste and smell of formula." | 13 (12%) |
|  | **Level of dietary restriction** | Mention of the extent of control over the quality and quantity of food that can be consumed. Example: "The fact that I often feel hungry after the diet." | 7 (6.4%) |
| **5. Factors related to the individual** | **Lack of information about PKU** | The perception of lack of information about health problems in the case of poor adherence to treatment and about the disease. Example: "Lack of information about the disease. | 3 (2.7%) |
|  | **Barriers to adherence due to other daily demands** | Mention of time and management difficulties in implementing the treatment. Example: "Lack of time to prepare new recipes". | 7 (6.4%) |
| Unclear responses, not allowing classification. | | | 5 (4.6%) |

*Note*. The items were translated from the Brazilian Portuguese to English to the present paper.

Therefore, this analysis suggested that 42.6% of the patients' responses were related to socioeconomic factors, 20.4% to team and health system factors, 23.1% to treatment factors and 9.25% to individual factors. There were no responses that were considered to be related to the disease factors dimension.

Table S2. Qualitative analysis of the 693 statements of caregivers/parents.

| **Dimensions according to WHO (2003)** | **Themes** | **Definition** | | | **Frequency**  **n (%)** |
| --- | --- | --- | --- | --- | --- |
| **1. Socio-economic factors** | **Distance to treatment center** | Mention of distance, need for transportation. Keywords: transportation, driving, far, near. Example: "Distance from reference centers". | | | 32 (4.6%) |
|  | **Barriers to access to special food for treatment** | Difficulties due  to cost | | Mention of the high price associated with food and diet products. Example: “High cost of allowed products.” | 91 (13.1%) |
|  |  | Difficulty finding or accessing food | | Mentioning difficulties in finding or accessing dietary foods. Example: "Difficulty finding foods suitable for phenylketonuria." | 86 (12.4%) |
|  |  | Limited variety of special food to purchase | | Perception of few options available for purchase. Example: "Few food options on the market." | 21 (3.0%) |
|  | **Lack of social support from community** | Difficulty finding PKU-appropriate meals at school | | Mention the lack of support to the child's needs in school. Example: "School and other places don't provide food as requested." | 12 (1.7%) |
|  |  | Lack of PKU-appropriate meal options in restaurants | | Lack of places that considered dietary restrictions. Example: "The lack of more specific options in bars, restaurants, and pizzerias." | 20 (2.8%) |
|  |  | Lack of support from social partners | | Perceived lack of support. Example: "More support from families. | 18 (2.5%) |
| **2. Factors related to the health team and health system** | **Health Team Support** | The importance of health team training | | Mention the lack of adequate education of professionals. Example: "Lack of information about the disease on the part of health professionals". | 26 (3.7%) |
|  |  | The importance of multidisciplinary care | | Mentioning the lack of specialists in different health areas in care. Example: "Better multidisciplinary hospital structure". | 15 (2.1%) |
|  | **Health System Assistance** | Quality aspects of available resources | | Difficulties related to operations in health system. Example: "Lack of communication with the dispensary that supplies the amino acid mixture". | 15 (2.1 %) |
|  |  | Quantity aspects of available resources | | Low number of appointments or blood tests locations. Example: "More frequent appointments. | 9 (1.2%) |
|  | **Free access to amino acid mixture (medical food)** | Problems getting a free amino acid mixture | | Difficulty getting the medical food regularly. Example: “Delivery of the amino acid mixture is no regular.” | 27 (3.8%) |
|  |  | It is not allowed to choose the medical food that is preferred by the patient | | Not having a choice of different types of amino acid mixtures. Example: "Patients lack the right to choose the amino acid mixture they want to consume." | 6 (0.8%) |
|  | **Information provided to the public** | Failure to educate the general public about PKU | | Perception of lack of disease information for general public. Example: "Better information about the disease to raise awareness among the public". | 17 (2.4%) |
|  |  | Lack of information on Phe in food products | | Lack of clarity on food labels, difficult to access or conflicting tables that indicate the amount of Phe according to each food. Example: "Clear information on product packaging". | 16 (2.3%) |
|  | **Government support** | Improving public assistance | | Mention the need for improvement in patient-care processes. Example: "Laws and legislation to support patients". | 9 (1.2%) |
|  |  | Helping with access to special food | | Mention of the lack of government assistance for the acquisition of low-protein foods. Example: “I think that in addition to the amino acid mixture, the government could offer, for example, some type of special flour for making bread and cakes.” | 18 (2.5%) |
|  |  | Access to adjuvant treatment medications | | Limited access to a drug that is useful in the treatment of some patients. Example: "Difficulty accessing sapropterin dichlorate, which would improve the patient's quality of life". | 9 (1.2%) |
|  |  | Financial assistance | | Mention lack of financial support for patients and caregivers. Example: "Financial assistance with treatment.” | 7 (1%) |
| **3. Disease- related factors** | Neurological impairment | Mention the health problems associated with delayed developing. | | | 1 (0.1%) |
| **4. Treatment-related factors** | Difficulty in consuming the amino acid mixture | Mention of low palatability and low quality of the provided medical food. Example: "Quality of amino acid mixture - taste and smell". | | | 72 (10.3%) |
|  | Perception of lack of availability of other medications to treat the disease | Mention of lack of other medications and more research. Example: “A specific medication for PKU, so that such a restrictive diet is not necessary.” | | | 21 (3.0%) |
|  | Level of dietary restriction | Mention of the small variety of foods allowed in the diet. Example: "Few food choices for the diet." | | | 48 (6.9%) |
|  | Difficulties with food preparation | Mention of difficulties in calculating and controlling the amount of phenylalanine in meals, having time to prepare meals, difficulty with recipes. Example: "Measuring the amount of protein consumed throughout the day". | | | 29 (4.1%) |
| **5. Individual-related factors** | Lack of information  about PKU | Lack of information about the disease and its treatment. Example: "What are the consequences of elevated phenylalanine levels?" | | | 10 (1.4%) |
|  | Patient developmental stages | Challenges related to the patient's age. Example: "It's more complicated in adolescence.” | | | 9 (1.2%) |
|  | Patient difficulty in acceptance of the disease, diet and restrictions | The patient's difficulty with diet. Example: "We have to take care of the child's psychology so he doesn't get frustrated." | | | 18 (2.5%) |
|  | **Emotions**  **of the caregiver** | Fear | Example: "Fear, insecurity, fear of not doing the best". | | 6 (0.8%) |
|  |  | Other negative feelings | Sadness, guilt, self-demand, overindulgence, loss of control over eating. Example: "Sometimes I feel sad because my children can't eat tasty foods." | | 9 (1.2%) |
| **Unclear responses, not allowing classification.** | | Unclear responses, not allowing classification.  Inaccurate responses, e.g., mentioning difficulty but not specifying it (n = 10)  Responses not related to the disease (n = 2) | | | 12 (1.7%) |
| **Responses not related to any of the five dimensions** | | Two responses mentioned the need for tools to facilitate daily treatment. And two responses mentioned the need for more PKU conferences and meetings. | | | 4 (0.5%) |

*Note*. The items were translated from the Brazilian Portuguese to English to the present paper.

The analysis suggested that 40.4% of the parents/caregivers’ responses were related to socioeconomic factors, 25.1% to team and health system factors, 24.5% to treatment factors and 7.5% to individual factors.

**Intersection between the 25 dichotomous items and the answers to the open question in the Perceived Barriers to PKU Treatment Inventory**

Considering the purpose of the last and optional item of the instrument, i.e. to identify barriers that were not covered by one or more of the 25 dichotomous items, the content recorded by the respondents indicated topics already included in the inventory and topics not covered. For example, the topic of "distance to treatment center" is already mentioned in item 24 of the instrument ("*The distance from my home to the hospital hinders my visits*."); the issue of "*lack of support from people in their social life*" is addressed in item 10 ("*I feel that I don't have people I can count on to help me follow my treatment*").

At the same time, other themes reported were not associated with a corresponding item in the instrument. For example, difficulties in accessing low protein foods, lack of appropriate PKU dietary options in school and work environments, lack of information about the disease for the general population, and others. Common to these themes were concerns that depend on changes beyond individual participation. The themes raised in the responses and the corresponding items in the inventory that deal with similar issues are shown in Table S3.

Table S3. Theme or subtheme identified in open-ended analysis and corresponding dichotomous item in the Perceived Barriers to PKU Treatment Inventory.

| **Theme or subthemes identified** | **Correspondent item (s)** |
| --- | --- |
| Patient acceptance of the disease, diet and restrictions. | 1, 2, 3, 8 |
| Lack of food intake out of home. | 4 |
| Lack of information about the condition. | 5,6 |
| Difficulties with food preparation. | 7,12,13,14 |
| Lack of support from social partners. | 10,11 |
| Difficulties in taking the metabolic formula. | 17 |
| The degree of dietary restriction. | 18 |
| Distance from treatment centre. | 24 |
| Problems getting free amino acid mixture. | 25 |
| Difficulty accessing low protein foods due to cost or availability in markets | - |
| Difficulty in accommodating food in the school or work environment | - |
| Lack of specialist training in the disease for the health care team. | - |
| Lack of greater involvement of psychologists and nutritionists in monitoring patients | - |
| Poor availability of government resources for blood testing and counselling | - |
| Lack of dissemination of information about the disease to the general population | - |
| Lack of clear information on Phe levels in food | - |
| Lack of government financial support for the purchase of industrial hypoprotein foods. | - |

*Note*. The items were translated from the Brazilian Portuguese to English to the present paper.
